# Cell density during differentiation can alter the phenotype of bone marrow-derived macrophages

**DOI:** 10.1186/2045-3701-3-30

**Published:** 2013-07-29

**Authors:** Chan Mi Lee, Jim Hu

**Affiliations:** 1 Physiology and Experimental Medicine, SickKids, 555 University Avenue, M5G 1X8, Toronto, Ontario, Canada; 2 Laboratory Medicine & Pathobiology, University of Toronto, 1 King’s College Circle, M5S 1A8, Toronto, Ontario, Canada

**Keywords:** Bone marrow-derived macrophages, Plating density, Macrophage phenotype

## Abstract

**Background:**

Bone marrow-derived macrophages (BMDMs) are widely used primary cells for studying macrophage function. However, despite numerous protocols that are currently available, lack of a notable consensus on generating BMDMs may obscure the reliability in comparing findings from different studies or laboratories.

**Findings:**

In this study, we addressed the effect of cell density on the resulting macrophage population. With reference to previously published methods, bone marrow cells from wild type C57BL/6 mice were plated at either 4 × 10^5^ cells or 5 × 10^6^ cells per 10 cm and cultured in 20% L-cell conditioned media for 7 days, after which they were analyzed for cell surface markers, production of proinflammatory cytokines, and responsiveness to polarizing signals. Reproducibly, cells plated at lower density gave a pure population of CD11b^+^F4/80^+^ macrophages (97.28 ± 0.52%) with majority being Ly-6C^-^Ly-6G^-^ and c-Fms^+^, while those plated at higher density produced less CD11b^+^F4/80^+^ cells and a considerably higher proportion of CD11b^+^F4/80^+^CD11c^+^ (68.72 ± 2.52%) and Ly-6C^-^Ly-6G^+^ (71.10 ± 0.90%) cells. BMDMs derived from higher plating density also secreted less proinflammatory cytokines such as IL-6, IL-12 and TNF-α and were less phagocytic, and had a different pattern of expression for M1- and M2-related genes upon LPS or IL-4 stimulation.

**Conclusions:**

Overall, our findings indicate that altering cell density during BMDM differentiation can give rise to distinct macrophage populations that could vary the outcome of a functional study.

## Findings

### Bone marrow-derived macrophages (BMDMs) are commonly used but methods are not conformed

Macrophages are phagocytic cells which play a crucial role in the first line of defense against pathogens and environmental toxins, and provide a link between innate and adaptive immune response. They express a wide range of Toll-like receptors (TLRs) and pattern-recognition receptors (PRRs) to detect endogenous danger signals and display an incredible plasticity, where their functions can be significantly and specifically altered by surrounding cytokines [1]. As a model system to study the function of this highly diversified and complex cell type, bone marrow-derived macrophages (BMDM) differentiated *in vitro* from bone marrow myeloid progenitors have enabled gene function studies in macrophages in general, as well as providing an abundant source of primary cells that are otherwise difficult to obtain. However, despite decades of application in a wide variety of experimental settings, there has not been a uniformly agreed BMDM culture method that is available to the scientific community. This notable lack of consensus on generating BMDMs may obscure the reliability in comparing findings from different studies or laboratories, as publications often do not provide detailed description of the procedures involved in the generation of primary cells.

### Culturing of bone marrow cells under different densities during *in vitro* differentiation

To generate BMDMs, the myeloid progenitor cells isolated from the bone marrow are cultured in the presence of macrophage colony-stimulating factor (M-CSF), which signals through its receptor M-CSFR (= c-Fms) to mediate monocytic lineage proliferation and differentiation [2,3]. M-CSF is now commercially available as a recombinant protein, or, more classically, from L-929 fibroblast cell line conditioned media. To test the reliability of utilizing different BMDM culture methods, we analyzed the effect of cell density during BMDM differentiation on the resulting macrophage population, as different plating density evidently varied between established protocols. With reference to previously published methods (such as [4-7]), bone marrow (BM) was flushed from femur and tibia of wild-type 9–12 week-old C57BL/6 mice, dispersed into single cell suspension after red blood cell (RBC) lysis and were plated at either 4 × 10^5^ cells or 5 × 10^6^ cells per 10 cm non-tissue culture-treated petri dishes in 10 mL of 20% L-929-conditioned media (20% L-cell conditioned media, 10% FBS, 2 mM L-glutaMAX, 10 IU/mL penicillin and 10 μg/mL streptomycin in 1× RPMI). On Day 3, additional 5 mLs of the growth media were added per petri dish and cells were harvested for assays on Day 7. The two cell populations were then analyzed for their expression of cell surface markers, secretion of inflammatory cytokines, phagocytosis, and expression of macrophage polarization genes. All animal work was performed in compliance with The Toronto Centre for Phenogenomics (TCP) Animal Care Committee (ACC)’s guidelines and approved Animal Use Protocol (AUP#0062).

### Macrophages grown under different cell densities express distinct cell surface markers

**Figure 1:**
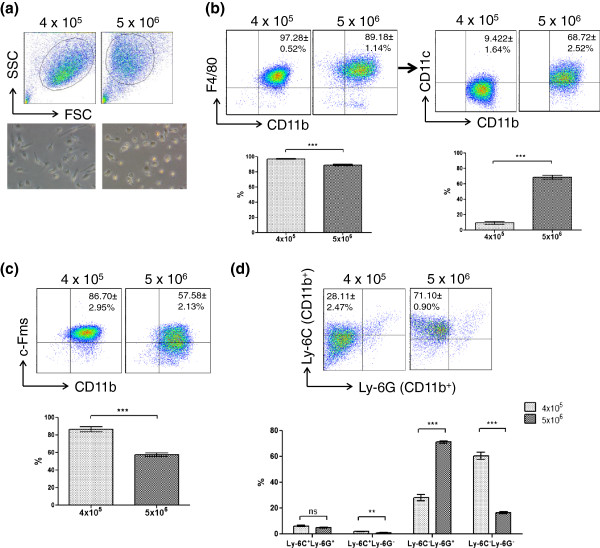
**Differentiation of bone marrow cells at different densities results in phenotypically distinct BMDM populations.** Plating cells at higher density produces **(a)** smaller and rounder macrophages, with top panel showing the forward-side scatter plot bottom panel showing the phase contrast images; **(b)** less CD11b^+^F4/80^+^ cells but more CD11b^+^F4/80^+^CD11c^+^; **(c)** less CD11b + c-Fms^+^; and **(d)** more CD11b^+^Ly-6G^+^ cells. For flow cytometric analysis, 2 × 10^5^ BMDM cells detached from culture dishes by 5 mM EDTA 1× PBS were stained with appropriate conjugated primary antibodies, using unstained cells and isotype antibodies as negative controls and Fixable Violet™ staining (Invitrogen) for dead cell exclusion. Data was acquired by BD LSRII flow cytometer and analyzed with Flowjo software. Results represent the mean (±SE) of three experiments of three independent samples each, and statistical analysis was done by student t test with Welch’s correction where appropriate. *** P < 0.0005; ** P < 0.005; * P < 0.05.

Flow cytometric analysis of monocytic/macrophage maturation markers of BMDMs generated under low (4 × 10^5^) or high (5 × 10^6^) plating concentrations revealed that the same bone marrow cells grown under different densities can give rise to phenotypically distinct populations (Figure 1). Reproducibly, cells plated at lower density gave a pure population of CD11b^+^F4/80^+^ macrophages (97.28 ± 0.52%) (Figure 1b) with majority being Ly-6C^-^Ly-6G^-^ (60.46 ± 2.88%) and c-Fms^+^ (86.70 ± 2.95%) (Figure 1c, d) which denotes a mature phenotype [8], while those plated at higher density produced less CD11b^+^F4/80^+^ (89.18 ± 1.14%) cells and a considerably higher proportion of CD11c^+^ (68.72 ± 2.52%) and Ly-6C^-^Ly-6G^+^ (71.10 ± 0.90%) cells (Figure 1b,d).

### Different macrophage differentiation conditions can lead to functional disparity

**Figure 2:**
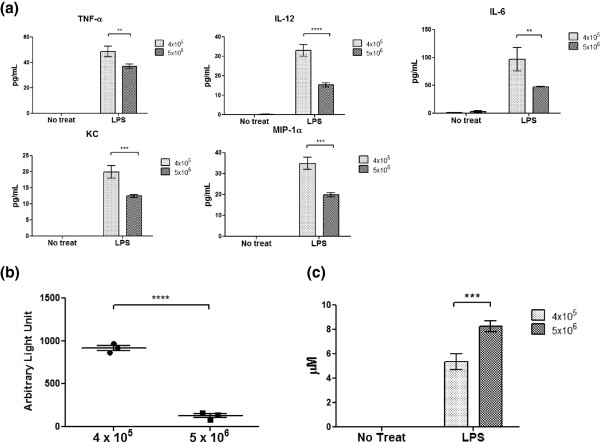
**Functional comparison of BMDMs differentiated under different plating densities. (a)** TNF-α, IL-6, IL-12, KC and MIP-1α were measured from culture supernatant using ELISA kits from Peprotech following the manufacturer’s instructions. Cells were plated on 6-well plates and stimulated with 100 ng/mL LPS (*Escherichia coli* serotype 0128:B12, Sigma) for 18 hours. **(b)** Phagocytosis assay was performed by plating 1x10^5^ cells on a 96-well plate in triplicates and incubating with fluorescein-tagged K-12 bioparticle (Invitrogen) for two hours and nonspecific signals were blocked with trypan blue. Fluorescence was detected on the Molecular Devices Spectra Max 190 microplate reader at 480 nm excitation and 520 nm emission wavelengths. **(c)** Presence of nitric oxide (NO) in the culture supernatant of the LPS-stimulated BMDM was measured colometrically using Greiss reagent (Molecular Probes) following the manufacturer’s instructions. Results represent three experiments of three biological samples each. Statistical analysis was done by one-way ANOVA with Bonferroni post-test. *** P < 0.0005; ** P < 0.005; * P < 0.05.

**Figure 3:**
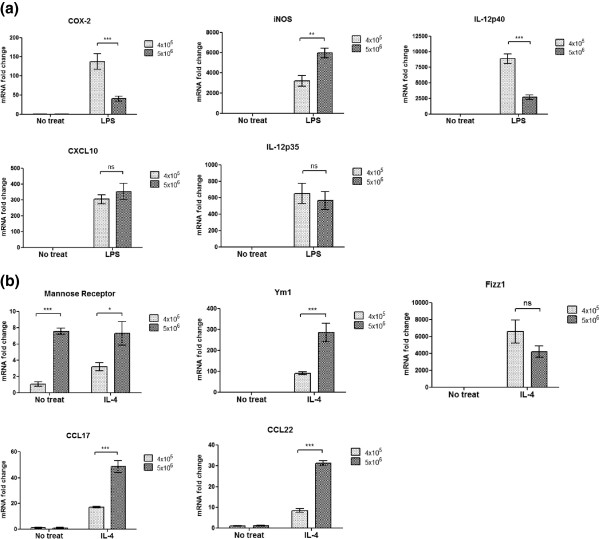
**Expression of macrophage polarization genes in BMDMs. (a)** Expression of genes related to the M1 polarization state upon activation with 100 ng/mL LPS for 18 hours and **(b)** M2 polarization state after treatment with 10 ng/mL of IL-4 (Peprotech) for 18 hours. Total RNA was isolated by GE RNAspin mini kit and reverse transcribed with Super Script II (Invitrogen) and run on ABI 7500 qPCR machine using ABI SYBERGreen Mastermix. Statistical analysis was done by student t test with Welch’s correction where appropriate. Result is a representative of three independent experiments with three biological samples per datapoint. *** P < 0.0005; ** P < 0.005; * P < 0.05.

To examine whether the observed phenotypic difference had a functional consequence, BMDMs grown under different densities were compared for their ability to secrete cytokines and phagocytose foreign materials. Noticeably, BMDMs derived from higher plating density secreted less proinflammatory cytokines such as TNF-α, IL-12, IL-6, KC and MIP-1α (Figure 2a) in response to LPS, were less phagocytic (Figure 2b), and had a different pattern of expression for M1- and M2-related genes upon LPS or IL-4 stimulation (Figure 3). It was interesting to note, however, that cells grown under higher plating density displayed increased nitric oxide (NO) production (Figure 2c), despite the fact that the same number of live cells (dead cells excluded by trypan blue staining) of each group were used for functional assays. In conclusion, our data indicate that BMDM density during *in vitro* differentiation can have a functional implication on the final macrophage populations, at least in BMDMs derived from C57BL/6 strain of mice.

### Discussion and Concluding Remarks

Cells grown at higher density expressed significantly higher levels of Ly-6G (=Gr-1) and showed a stronger tendency towards M2 polarization upon IL-4 treatment (Figure 3b). Given that CD11b^+^Ly-6C^-^Ly-6G^-^ represent a mature BMDM [8], these cells may be deemed as ‘less mature’ and points to the possibility that the higher proportion of Ly-6G^+^ cells resulted from insufficient supply of M-CSF in the growth medium. However, the M-CSF:BM cell ratio required for full BMDM maturation is currently unknown, and published studies typically use 10-30% L929-conditioned media or 20–100 ng/mL recombinant M-CSF over a period of 5–8 days. The possibility that M-CSF fell short during differentiation of cells cultured at 5 × 10^6^ density may be tested by growing a fixed number of BM cells under different known concentrations of recombinant M-CSF. Morphologically, BMDMs matured under higher density tended to be smaller and rounder (Figure 1a), despite the fact that the overall final cell density on Day 7 was similar between the two groups of macrophages. It is thus questionable whether bone marrow stromal cells that eventually undergo apoptosis during the *in vitro* monocytic differentiation might have contributed to this effect. One way to test the effect of stromal cells in BMDM maturation would be to co-culture bone marrow stromal cell line such as M2-10B4 with the myeloid progenitor cells at different ratios and characterizing the final BMDM population. In regards to the relationship between the maturation states of a monocytic cell to their polarization propensity, an earlier study had indicated that immature macrophages secrete more IL-12 [9]. However, a maturation status of a macrophage might be more complex than just defined by cell surface markers or morphology. Myeloid-derived suppressor cells (MDSCs), which are known to be CD11b^+^Gr-1^+^ in mice for example, have been demonstrated to undertake an immunosuppressive role in cancer, but monocytic MDSCs produce high levels of NO [10], which is reflective of characteristics displayed by the CD11b^+^Ly-6C^-^Ly-6G^+^ BMDMs in this study. Therefore, this calls for better understanding of monocytic maturation steps and their corresponding function and the environmental factors that might influence the course of maturation, as well as for a commonly established protocol for BMDM generation to ensure consistency between studies.

## Abbreviations

BMDM: Bone marrow-derived macrophages; BM: Bone marrow; RBC: Red blood cell; M-CSF: Macrophage colony-stimulating factor; M-CSFR: Macrophage colony-stimulating factor receptor; LPS: Lipopolysaccharide; MDSC: Myeloid-derived suppressor cell.

## Competing interests

Both authors claim no competing interests in regards to this project.

## Authors’ contribution

CML conceived and executed the experimental procedures and drafted the manuscript. JH supervised the project. Both authors read and approved the final manuscript.
